# Prophylaxis of Epidemic of Puerperal Fever

**Published:** 1852-06

**Authors:** M. Semmeliveis


From the Charleston Medical Journal.

*Prophylaxis of Epidemic of Puerperal Fever.* By M. SEMMELIVEIS, (*Gazette Medicale*, Jan. 1851.)

M. SEMMELIVEIS, who is a physician accoucheur to the great Maternite Hospital of Vienna, has recently brought overwhelming testimony in proof of the communicability of this dreadful disease from patient to patient through the medium of the physicians or attendants.

The edifice was erected in 1784, and as its wards were always encumbered with women in labor, an addition was made to it in 1838; but only separated from the old building by a thin partition, with doors and windows for communication. After this arrangement was made, the *old* clinique was held in the *new* building, and the *new* clinique in the *old* building. At first, the mortality from puerperal fever which had, all along, been frightful, was about equal in the two cliniques. In 1839, by a decree of the government, one clinique (the last established) was abolished, and only the female students of midwifery admitted to it; the male students being restricted to the first clinique. In a few years, the difference in the mortality of the two divisions of the hospital became remarkable, so much so as to cause the appointment of a committee by the government, to investigate the causes of the greater rate in the one than in the other. The bad washing of the bed linen, the unfavorable situation of the wards of the new building, etc., etc., were suspected of playing a part in the increase of the number of deaths, but that notion was soon abandoned as untenable. After a protracted study of the probable causes, M. Semmeliveis conceived the idea that it resided in the circumstance that the male students were occupied the whole time, alternately in making dissections of the bodies of those that had died of puerperal fever, and in delivering the women, while the female students, who attended the other division, never went into the dissecting rooms. M. Semmeliveis, convinced that it was by innoculation, that is, by the application of the morbid products, solid or fluid, of the dead body to the genital parts of the parturient female, that the disease was propagated, devoted himself to a study of the means of preventing it.

Knowing the difficulty, if not the impossibility, of removing from the hands the cadaveric odor (which is indicative of the retention of some morbid atoms,) with soap, etc., by an extended series of experiments, he found that a solution of chloride of lime was effectual. It was therefore ordered that every student, before being admitted to parturient women, should wash his hands in the above solution, and use the nail brush with great care. This precaution was sufficient to ensure, in a short time, a great reduction in the mortality, from ten to fifteen, as it formerly was, to one per cent.; the rate being now about equal in the two divisions of the institution.

